# Prevalence of clarithromycin-resistant *Helicobacter pylori* strains in gastric mucosa-associated lymphoid tissue lymphoma patients

**DOI:** 10.1007/s00277-016-2672-4

**Published:** 2016-04-19

**Authors:** Ceren Bilgilier, Ingrid Simonitsch-Klupp, Barbara Kiesewetter, Markus Raderer, Werner Dolak, Athanasios Makristathis, Christoph Steininger

**Affiliations:** Department of Internal Medicine I, Division of Infectious Diseases and Tropical Medicine, Medical University of Vienna, Währinger Gürtel 18-20, 1090 Vienna, Austria; Clinical Institute of Pathology, Medical University of Vienna, Vienna, Austria; Department of Internal Medicine I, Division of Oncology, Medical University of Vienna, Vienna, Austria; Department of Internal Medicine III, Division of Gastroenterology and Hepatology, Medical University of Vienna, Vienna, Austria; Department of Laboratory Medicine, Division of Clinical Microbiology, Medical University of Vienna, Vienna, Austria

**Keywords:** *Helicobacter pylori*, Gastric mucosa-associated lymphoid tissue lymphoma, Antimicrobial resistance, Clarithromycin, *cagA*

## Abstract

Gastric MALT lymphoma is closely associated with *Helicobacter pylori* infection. Bacterial eradication therapy comprising clarithromycin is the first-line treatment in gastric MALT lymphoma patients. However, antimicrobial resistance to clarithromycin has been increasing in Europe, and thus far, it has not been examined in gastric MALT lymphoma patients. Based upon histopathological investigation, 17 adult gastric MALT lymphoma patients were identified to be related with *H. pylori* infection between 1997 and 2014. Detection of *H. pylori* infection in these patients and clarithromycin susceptibility testing were performed by 23S rRNA gene real-time PCR. Twelve of the patients were confirmed with *H. pylori* infection by real-time PCR. Among these patients, only two were found to be infected with clarithromycin-resistant *H. pylori* strain. In one of them, both the clarithromycin-resistant and sensitive genotype were detected. The rate of clarithromycin resistance was 15.4 %. Clarithromycin resistance pattern in gastric MALT lymphoma patients is under the predictions since a previous study performed in Central Europe revealed a rate of 36.6 % in Austria. Considering the low antimicrobial resistance rate, clarithromycin is still an option in gastric MALT lymphoma management.

## Introduction

Extranodal marginal zone lymphoma of mucosa-associated lymphoid tissue (MALT) lymphoma most commonly affects the stomach where it accounts for almost 50 % of all gastric lymphomas [1–3]. Still, gastric MALT lymphoma is a rare disease and therapeutic recommendations are mostly based on retrospective data and clinical experience, while only few randomized controlled data are available [4].

Current evidence strongly supports a causal role of *Helicobacter pylori* infection in the evolution of the disease, and antibiotic treatment is the therapeutic mainstay of management of such patients. In almost 90 % of cases, gastric MALT lymphoma is thought to be associated with infection with this microaerophilic, Gram-negative bacterium, although recent studies have shown a much higher rate of *H. pylori*-negative patients [4–6]. We could confirm recently this trend in an Austrian cohort by observing a rate of *H. pylori*-negative cases of MALT lymphoma of 32 % [7]. Still, patients with gastric MALT lymphoma are significantly more likely to have evidence of previous *H. pylori* infection than matched controls [8]. Eradication of *H. pylori* infection is associated with regression of gastric MALT lymphoma in the large majority of patients and has been reported to be beneficial even in patients without laboratory confirmation of *H. pylori* infection [3, 7, 9]. The most widely accepted hypothesis for the pathogenesis of gastric MALT lymphoma states accordingly that *H. pylori* infection of the stomach indirectly stimulates B cells by activation of CD4^+^ T cells and also CagA is translated into lymphoma cells [10, 11]. Consecutively, a chronic activation of B cells and local inflammation with the generation of free reactive oxygen species triggers the evolution of gastric MALT lymphoma [11].

Accordingly, *H. pylori* eradication is recommended by all current international guidelines as first-line treatment for patients suffering from gastric MALT lymphoma, irrespective of stage [2, 3, 12]. The standard treatment regimen recommended for *H. pylori* eradication consists of a clarithromycin-based triple therapy, also including a proton pump inhibitor (PPI) twice a day and amoxicillin or metronidazole for 7 or 14 days [3]. Nevertheless, antimicrobial resistance rates have been increasing dramatically in clinical *H. pylori* strains. In Europe, resistance rates to the key antimicrobial clarithromycin range between 5.6 and 36.6 %, with Austria leading the table [13]. Evaluation of clinical *H. pylori* strains for antimicrobial resistance is recommended for patients with treatment failures, as well as those originating from geographic regions with high rates of antimicrobial resistance [4]. However, in gastric MALT lymphoma patients, bacterial culture has a clearly lower sensitivity than histology even if performed under optimized conditions [14], thereby limiting the utility of data regarding antimicrobial resistance. As a consequence, the optimum antimicrobial combination treatment in gastric MALT lymphoma patients from geographic regions with high resistance rates remains yet to be defined.

In this study, we therefore evaluated genotypic clarithromycin resistance rates of clinical *H. pylori* strains in a cohort of gastric MALT lymphoma patients through utilizing a highly sensitive and reliable real-time PCR assay. We found that clarithromycin resistance rates are clearly lower in gastric MALT lymphoma patients than anticipated from epidemiological information in the general population.

## Materials and Methods

### Patients

Patients eligible for this retrospective, monocentric analysis had a histological confirmation of gastric MALT lymphoma diagnosed between January 1997 and December 2014. In addition, only gastric biopsy samples that were histologically positive for *Helicobacter*-like organisms (HLO) were included in the present study. The presence of *H. pylori* could be further confirmed with use of VENTANA anti-*H. pylori* (SP48) rabbit monoclonal primary antibody (Roche Diagnostics, Basel, Switzerland). Only cases with sufficient archived primary tumour tissue were selected. Demographic and clinical data were collected retrospectively from patients’ notes and prescription charts. The study was performed in accordance with the Declaration of Helsinki and good clinical practice guidelines and was approved by the local ethics committee (EK1548/2014). Informed consent was obtained for the diagnostic procedures from all individual participants included in the study.

### DNA purification

Genomic DNA was extracted from representative formalin-fixed, paraffin-embedded tissue blocks of biopsy samples collected from gastric MALT lymphoma patients. DNA was purified using an EZ1 DNA Tissue kit (No. 953034; Qiagen, Hilden, Germany) according to the tissue protocol followed by automated purification using the EZ1 instrument.

### Microbiological evaluation

All HLO-positive biopsy samples available from the present gastric MALT lymphoma patients were tested for the presence of *H. pylori*-specific DNA with use of a previously described assay [15]. In brief, this PCR assay amplifies a *H. pylori* DNA fragment of 137 bp of the 23S rRNA gene including the sites of mutation that confer phenotypic resistance to clarithromycin. Isolated DNA was subjected to 70 amplification cycles (with a temperature transition rate of 20 °C/s) of denaturation at 95 °C for 5 s, annealing at 65 °C for 10 s, and primer extension at 72 °C for 6 s. The limit of detection of this PCR assay for pure *H. pylori* DNA was 4 fg per PCR, which is equivalent to 2.2 bacteria and *H. pylori* specificity and sensitivity of this PCR assay was confirmed to be 98 and 100 %, respectively. Genotypic clarithromycin resistance of clinical *H. pylori* strains was evaluated with use of melting point analysis performed after the amplification of the 23S rRNA gene as described previously. For this purpose, a probe binding to the site of mutations in the 23S rRNA gene was used during amplification. Melting temperatures were analysed for significant differences to the wild-type *H. pylori* genotype (the melting temperature is lower in mutated strains than in wild type, clarithromycin-sensitive strains because of the mismatch between the amplicon and the probe, which corresponds to the wild-type susceptible genotype). Every PCR run included several positive and negative controls. As positive controls, DNA samples extracted from pure clarithromycin-sensitive and resistant *H. pylori* isolates were used. As negative control, master mix solution filled up with water to the final volume was applied.

PCR amplification of the *cagA* variable region was performed as previously described by using the primers and conditions with some modifications [16]. Primers were modified to account for the mismatches at primer binding sites among the different *H. pylori cagA* genotypes available through GenBank (Table 1). Alignment of sequences was done using CLC Genomics Workbench Software (CLC bio, QIAGEN, Hilden, Germany). First screening of samples was done with use of the EPIYA PCR using the forward primer CagA-Tfw and three reverse primers (CagA-rvP1, CagA-rvP2, and CagA-rvP3) to amplify the three different EPIYA sites of the *cagA* gene, P1, P2, and P3, and thereby determine the EPIYA motifs encoded. To exclude a low sensitivity of this PCR assay as possible explanation for negative test results, a second *cagA*-specific PCR that amplifies the entire 3′ repeat regions of the *cagA* gene was performed using the forward primer CagA-Tfw and reverse primer CagA-Trv. In present study, iProof DNA polymerase (Bio-Rad Laboratories, Hercules, CA) was used, which has the 3′ to 5′ exonuclease proofreading activity and may be more accurate than Taq polymerase. A reaction mixture containing 0.2 mM of each deoxynucleoside triphosphate, 3 mM MgCl_2_, 0.5 μM of both forward and reverse primers, 3 % DMSO, 0.02 U of iProof DNA polymerase/μl, 1× iProof HF Buffer (Bio-Rad Laboratories, Hercules, CA), and 1 μl of DNA was used. The mixture was incubated at 98 °C for 30 s, followed by 35 cycles at 98 °C for 10 s, 57 °C for 15 s, and 72 °C for 15 s, and a final extension at 72 °C for 5 min. The resulting PCR products were analysed on 2 % agarose gel.

**Table 1:** Primers used for *cagA* amplification

Primer	Sequence (5′-3′)
CagA-Tfw	GGAACCCTAGTCGGTAATG
CagA-Trv	ATCTTTGAGMTTGTCTATCG
CagA-rvP1	GTCCTGYTTTCTTTTTATTAACYTKAGC
CagA-rvP2	TTTAGCAACTTGAGYRTAAATGGG
CagA-rvP3	ATCAATYGTAGCATAAATGGG

(M=A+C, Y=T+C, K=G+T, and R=G+A)

## Results

From January 1997 to December 2014, a total of 97 patients with gastric MALT lymphoma were evaluated and treated at Vienna General Hospital, Austria as previously described [7]. A total of seven patients (7 %) had detectable HLO in gastric biopsies at the time of evaluation. In one patient, HLO could be detected in three samples collected over a period of 3 years. In addition, we also evaluated gastric biopsy samples collected from additional ten patients who were treated at other institutions over the same period and whose samples were sent to our institution for histological evaluation. HLO were detected in all of these ten samples as well. The mean age (±SD) of the 17 patients was 72 ± 9 years (range, 57–88 years) and 71 % (12 of 17) of them were male. Remarkably, seven (41 %) of the 17 MALT lymphoma patients received eradication therapy for *H. pylori* before the present detection of HLO in their gastric biopsies.

Histological evaluation further confirmed the presence of signs of gastritis in all 19 gastric biopsy samples from the 17 patients. Semi-quantitative evaluation of HLO density in the samples showed low density in eight, intermediate density in six, and high density in five samples.

Isolation of DNA from formalin-fixed tissue samples and testing with the *H. pylori*-specific PCR assay yielded positive test results in 14 (74 %) of the 19 histologically HLO-positive samples. These 14 *H. pylori*-positive samples were collected from 13 different gastric MALT lymphoma patients. A false-negative PCR result was not associated significantly with a low HLO density in the gastric biopsy samples (4/8 vs 1/11; *P* = 0.111 [Fisher Exact]). Analysis of the 23S rRNA gene for genotypic clarithromycin resistance revealed sensitive strains in 11 (79 %) of the 14 samples. Clarithromycin-resistant *H. pylori* strains could be detected only in two different samples from a single patient and in the sample from a further patient, in which a mixed genotype was detected. The rate of clarithromycin-resistant *H. pylori* infections was accordingly 15 % (2 of 13).

Follow-up studies revealed that *H. pylori* infection was eradicated successfully in all five patients with information available. Remarkably, the single patient with repeated detection of a clarithromycin-resistant *H. pylori* strain was eradicated successfully between these positive test results as indicated by a negative histological evaluation of gastric biopsies. Upon follow-up gastric biopsies, however, the patient’s samples were again positive for *H. pylori* indicative for reinfection by a clarithromycin-resistant *H. pylori* strain. Eight patients were lost to follow-up due to gastrectomy (*n* = 1), an additional malignancy requiring immediate chemotherapy or rapid progression of MALT lymphoma (*n* = 3), or unknown reasons (*n* = 4).

In addition, we tested the gastric biopsy samples for the presence of the *cagA* because *H. pylori* strains possessing that gene are more likely to cause disease than those lacking it [17–19]. All samples tested by EPIYA PCR were negative for the *cagA*. To avoid a bias by a limited sensitivity of this PCR, we tested all samples also with a more sensitive pan-*cagA* PCR with the same results.

## Discussion

Antimicrobial eradication treatment is the mainstay in the management of gastric MALT lymphoma patients based on the recognition of a likely causal role of *H. pylori* infection in the evolution of the disease and excellent response rates to antimicrobial combination treatment [7, 20]. *H. pylori* antimicrobial resistance rates, however, increased clearly in the last decade with clarithromycin resistance rates as high as 37 % [13]. Contrary to our initial expectation, we found that clarithromycin resistance rates were as low as 15 % in the present cohort of gastric MALT lymphoma patients which suggests that clarithromycin is still an effective choice in the empiric eradication of *H. pylori* infection.

The standard antimicrobial regimen recommended for *H. pylori* eradication in the general population as well as in patients with gastric MALT lymphoma is based on a clarithromycin-backbone substituted with a PPI and amoxicillin or metronidazole for 7 or 14 days [3]. The increase in antimicrobial resistance, particularly in Central Europe, required a switch to second-line regimens in empiric eradication therapy [21]. Second-line treatments, however, are associated with a higher pill burden, higher rates of adverse events, and the risk to induce antimicrobial resistance in other infectious pathogens. In view of the limited information available on *H. pylori* antimicrobial resistance patterns in patients with gastric MALT lymphoma, data from the general population suggest that second-line regimens might also be required in the case of these patients. Our analyses, however, reveal a clarithromycin resistance rate as low as 15.4 %, which is clearly lower than expected from the high resistance rate of 37 % observed previously in Austria. Although the present observations have to be interpreted with caution due to the small size of the cohort studied, first-line antimicrobial eradication regimens still appear to be effective options for empiric treatment.

To determine the presence of *H. pylori* infection in gastric MALT lymphoma patients, a combination of several diagnostic techniques is advised by international guidelines, including invasive (e.g., histology, rapid urease test, or bacterial culture) and non-invasive techniques (e.g., serology and urea breath test) [2]. Establishing the diagnosis of *H. pylori* infection in gastric MALT lymphoma patients, however, may be difficult. Results obtained through the use of histology, bacterial culture, PCR, and serology are concordant only in 63 % of cases [14]. For the detection of *H. pylori* in gastric MALT lymphoma patients, bacterial culture of gastric biopsy samples was found to be unsatisfactory, when the generally high sensitivity of bacterial culture for the detection of infectious pathogens is considered, in a diversity of clinical samples [14].

Prevalence of *H. pylori* infection would be underestimated if only one diagnostic tool would be used. Still, we could identify HLO with use of histology only in seven of 97 gastric biopsy samples. The very small number of gastric MALT lymphoma patients with detectable HLO in their gastric biopsy samples requires explanation. Information on the prevalence of HLO positivity in gastric MALT lymphoma patients is still limited to a few case series [14]. In a large European cohort study, histology was positive for HLO in 29 of 90 patients with gastric MALT lymphoma. The present retrospective study was conducted at a tertiary hospital, and we may not exclude selection biases deriving from previous eradication therapies prescribed before referral, as well as a trend towards evaluation and management of more advanced stages of the disease [7]. Accordingly, it may currently be only hypothesized on the true prevalence of *H. pylori* positivity in gastric MALT lymphoma patients when direct diagnostic assays are used.

The presence of *H. pylori* in gastric biopsy samples could be confirmed by PCR in 74 % of samples that were HLO positive by histology. The PCR assay used was thoroughly evaluated and is highly sensitive [15] but, in the present study, was used for the detection of *H. pylori*-specific DNA in formalin-fixed, paraffin-embedded tissue samples. Formalin treatment also fragments DNA and traps it by extensive cross-linking between nucleic acid strands [22]. Moreover, the likelihood of a positive PCR assay did not correlate with the density of *H. pylori* in the tissue samples, which points rather to a technical than to a biological explanation for negative PCR results. DNA isolated from formalin-fixed, paraffin-embedded tissue samples is, therefore, of poor quality overall, and pose a major challenge for the detection of specific DNA targets.

Nevertheless, negative test results obtained with use of the *H. pylori*-specific PCR assay may also be explained by infection with *Helicobacter heilmannii*. A minority of gastric MALT lymphomas is associated with infection by *H. heilmannii*, which is difficult to differentiate from *H. pylori* when using standard stains in histology. Nevertheless, we also evaluated PCR-negative tissue samples with use of *H. pylori*-specific immunohistochemistry and found that the HLO detected were actually *H. pylori*. Accordingly, negative PCR results obtained from evaluation of formalin-fixed, paraffin-embedded tissue samples have to be interpreted with caution.

The evolution of gastric MALT lymphoma has been related specifically to *H. pylori* strains expressing the *cag* pathogenicity island, which is an important virulence factor in this infection [17]. WHO and International Agency for Research on Cancer define *cagA* as a human class I carcinogen [23, 24]. Upon *H. pylori* infection, CagA is translocated into gastric epithelial cells where it deregulates intracellular signalling pathways and thereby promotes lymphomagenesis [10]. *H. pylori* strains that do not encode *cagA* were associated with resistance to metronidazole, but not to clarithromycin [25–27]. *CagA*-specific antibodies are found in 89–96 % of sera from patients with gastric MALT lymphoma [18, 19]. In a previous study, the *cagA* gene was detected in 47 % of *H. pylori* strains from gastric MALT lymphoma patients [14]. In contrast, we were not able to identify a single patient harbouring a *cagA*-encoding *H. pylori* strain. The negative test results may again be attributable to the low quality of DNA recovered from formalin-treated biopsy samples. Still, we used two different and sensitive PCR assays to avoid false-negative test results [28]. A significant difference to the previous study, however, is the material used; we used primary samples whereas cultured bacteria were used in the previous study.

In conclusion, the antimicrobial resistance to clarithromycin in *H. pylori* strains obtained from gastric MALT lymphoma patients is relatively low and clarithromycin is still a valid choice in empiric eradication treatment. The presence of clarithromycin-resistant *H. pylori* strains, however, should be considered in patients with treatment failure.
